# In-Fiber Collimator-Based Fabry-Perot Interferometer with Enhanced Vibration Sensitivity

**DOI:** 10.3390/s19020435

**Published:** 2019-01-21

**Authors:** Bin Du, Xizhen Xu, Jun He, Kuikui Guo, Wei Huang, Fengchan Zhang, Min Zhang, Yiping Wang

**Affiliations:** 1 Key Laboratory of Optoelectronic Devices and Systems of Ministry of Education and Guangdong Province, College of Optoelectronic Engineering, Shenzhen University, Shenzhen 518060, China; dubin2016@email.szu.edu.cn (B.D.); 2166190111@email.szu.edu.cn (X.X.); 2150190116@email.szu.edu.cn (K.G.); huangwei2017@email.szu.edu.cn (W.H.); 2014170094@email.szu.edu.cn (F.Z.); zhangmin@szu.edu.cn (M.Z.); ypwang@szu.edu.cn (Y.W.); 2 Guangdong and Hong Kong Joint Research Centre for Optical Fibre Sensors, Shenzhen University, Shenzhen 518060, China

**Keywords:** fiber vibration sensor, graded index fiber, Fabry-Perot interferometer

## Abstract

A simple vibration sensor is proposed and demonstrated based on an optical fiber Fabry-Perot interferometer (FPI) with an in-fiber collimator. The device was fabricated by splicing a quarter-pitch graded index fiber (GIF) with a section of a hollow-core fiber (HCF) interposed between single mode fibers (SMFs). The static displacement sensitivity of the FPI with an in-fiber collimator was 5.17 × 10^−4^ μm^−1^, whereas the maximum static displacement sensitivity of the device without collimator was 1.73 × 10^−4^ μm^−1^. Moreover, the vibration sensitivity of the FPI with the collimator was 60.22 mV/g at 100 Hz, which was significantly higher than the sensitivity of the FPI without collimator (11.09 mV/g at 100 Hz). The proposed FPI with an in-fiber collimator also exhibited a vibration sensitivity nearly one order of magnitude higher than the device without the collimator at frequencies ranging from 40 to 200 Hz. This low-cost FPI sensor is highly-sensitive, robust and easy to fabricate. It could potentially be used for vibration monitoring in remote and harsh environments.

## 1. Introduction

The optic fiber sensors based on Fabry-Perot interferometers (FPIs) [1,2,3,4,5,6,7] have been attracting significant attention into broad range of physical [8], chemical [9,10], and biomedical [1,3,11,12] fields in recent years, due to their unique advantages, such as compact size [8,9,13], easiness for integration [6,14], immunity to electromagnetic interference [7], and resistance to high temperatures [8]. The intensity changes in their interference spectra, caused by small spatial displacements [6,15,16], makes these sensors ideal for detecting vibrations with high accuracy. Such sensitivity is critical for a variety of applications, for instance, earthquake or ocean wave monitoring, structural health monitoring for high voltage transformer, and hot fluids monitoring in aerospace engineering [17].

Several studies have reported a wide range of techniques and materials for fabricating vibration sensors [13,18,19,20,21,22]. For example, Lopez-Higuera et al. proposed a mechanical vibration sensor based on a FPI with a cantilever structure, with a sensitivity of 72 mV/g at 30 Hz [19]. This device could operate in a measurement range of 0.2–140 Hz, but it exhibited a complex structure and required accurate installation for proper functionality. Wu et al. reported a vibration sensor based on an optical fiber FPI. The device is fabricated by using polydimethylsiloxane (PDMS), and the sensitivity is 0.088 mV/mPa at 200 Hz [13]. However, PDMS cannot work at high temperatures. Moreover, Nishino et al. proposed a displacement and vibration sensor based on an intrinsic fiber-optic loop [15]. However, the output signal included very high harmonic components. Li et al. demonstrated a vibration sensor based on a diaphragm-type fiber Bragg grating (FBG) with a functional range of 10–150 Hz. The sensitivity of this structure is 31.25 pm/g at 100 Hz [21]. Subsequently, Li et al. reported a vibration sensor based on ultra-weak FBGs with a frequency response range from 10 to 1000 Hz [22]. Wang et al. presented a torsional vibration sensor based on a fiber Bragg grating with sensitivity of 0.3603 pm/(rad/s^2^) [23]. In most cases, the fabrication of FBGs requires additional refractive index modulation using costly elaborate hardware.

This study proposes a vibration sensing system, and a simple low-cost vibration sensor is a critical component. The device is based on a cantilever structure. It consists of a fiber FPI and an in-fiber collimator. This collimator was used to increase the fringe visibility and reduce the insertion loss of an FPI [6]. The sensor consists of a quarter-pitch graded index fiber (GIF) spliced with a hollow-core fiber (HCF), interposed between two single mode fiber (SMF) segments. The air cavity between the end faces of GIF and SMF acts as the FPI cavity. The transmission loss was reduced by use of optimal fiber fusion parameters. Simulated results suggested these sensors could be used to accurately detect weak vibration signals. The experimental response of this sensor is demonstrated and discussed.

## 2. Vibration Sensor System

**Figure 1:**
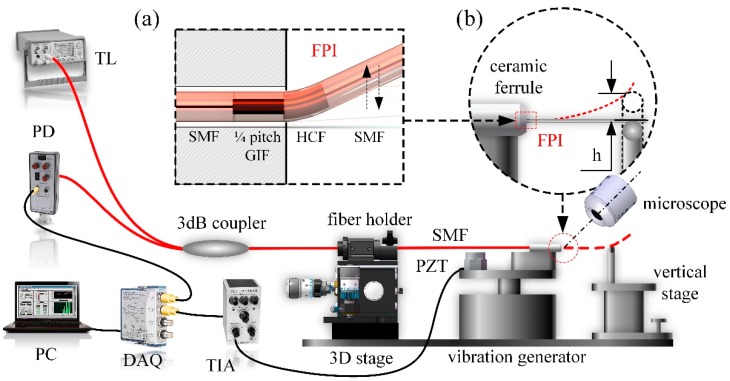
A schematic diagram of the proposed vibration sensor system (TL: tunable laser; FPI: Fabry-Perot interferometer; 1/4 pitch GIF: a quarter pitch graded index fiber (fiber collimator); SMF: single mode fiber; HCF: hollow-core fiber (air cavity of the Fabry-Perot interferometer); PD: photodiode; PZT: piezoelectric transducer; TIA: transimpedance amplifier; DAQ: data acquisition board; PC: personal computer). Insets (**a**) and (**b**) show the FPI sensor structure and the detailed schematic of the FPI sensor with a lateral displacement, respectively.

We proposed a novel, simple, vibration sensor system based on the all-optical fiber FPI cavity shown in Figure 1. The FPI with a fiber collimator is presented in the inset (a) of Figure 1. 

This sensor includes a quarter-pitch GIF, an HCF and two SMFs. A tunable laser (Agilent, 81940A, Palo Alto, CA, USA) was connected with the fiber sensor through a 1 × 2 3-dB coupler. The fiber sensor was fixed on a fiber holder and translated into the ceramic ferrule horizontally. Motion ceased when the FPI cavity section (the sensor) emerged from the front end of the ceramic ferrule. These components were mounted on a vibration generator while the fiber holder was mounted on a three-dimensional stage. A vertical stage and a narrow fulcrum bar were used to control the vertical displacement of the sensor, with its side-view image monitored by an optical microscope, as shown in the inset (b) of Figure 1. A PIN photodiode (New Focus, Model 2053, Irvine, CA, USA) was used as a power detector, converting the optical signal into an electrical signal. Moreover, a piezoelectric transducer (PZT) was placed along the fiber sensor to receive and record the vibration intensity. Subsequently, a transimpedance amplifier (TIA) was adopted to convert the charge signal generated in PZT into an amplified voltage signal. The analog voltage signal was then digitalized using a data acquisition board (National Instruments, cDAQ-9174 and 9215, Austin, TX, USA) and sent to a personal computer (PC) for the following signal processing.

## 3. Principle of Operation

**Figure 2:**
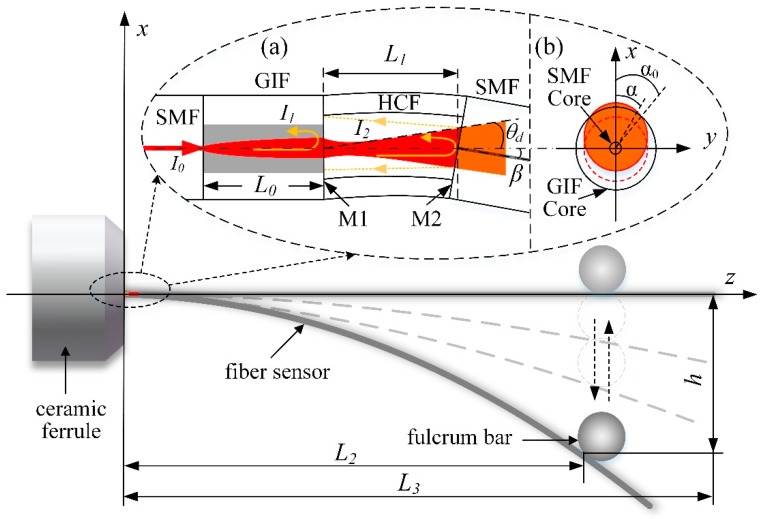
The working principle of the proposed FPI vibration sensor. Insets (**a**): a schematic of the proposed FPI; (GIF: graded index fiber; HCF: hollow-core fiber, SMF: single mode fiber) (**b**): the beam profile on M1.

The working principle for the proposed FPI vibration sensor is shown in Figure 2. As the sensor is deflected by applied force, an angle β is generated between the optical axis and the normal line of mirror 2 (M2). The wave front phase of the light reflected from the two mirrors (M1 and M2) will vary, producing changes in the spatial position and total intensity of the light. In addition, the photodiode will only detect the light reflected back into the core of the SMF. 

### 3.1. Divergence Angle of the Fiber Collimator

We analyzed the divergence angle of the quarter pitch GIF. Light propagates along an approximately sinusoidal path in the GIF [24]. The focusing parameter (*G*) of the GIF is defined as [24]:

(1) $$G=\frac{\sqrt{2\Delta }}{r},$$

where Δ is the relative refractive index difference between the GIF core and cladding, and r is the radius of the GIF core. Hence, the quarter-pitch of the GIF is calculated by [25]

(2) $$L_{0}=\frac{p}{4}=\frac{1}{4}\cdot \frac{2\pi }{G}=\frac{\pi }{2G}.$$

Here, P is the pitch length of GIF [24]. The mode field radius (ω) at the end surface of the quarter-pitch GIF can be expressed as [24]:

(3) $$\omega =\frac{\lambda }{G\cdot \pi \cdot n_{0}\cdot \omega_{0}},$$

where λ is the wavelength of light beam, $n_{0}$ is the refractive index along the GIF center axis, and $\omega_{0}$ is the mode field radius in SMF. The divergence angles of SMF $\theta_{d0}$ and a quarter-pitch GIF $\theta_{d}$ are obtained as [26]:

(4) $$\begin{array}{l} \theta_{d0}=\frac{\lambda }{\pi \cdot \omega_{0}},\\ \theta_{d}=\frac{\lambda }{\pi \cdot \omega }=G\cdot n_{0}\cdot \omega_{0}. \end{array}$$

when Δ = 0.02, r = 31.25 μm, $\omega_{0}$ = 5 μm (in SMF), λ = 1550 nm and $n_{0}$ = 1.491, the quarter-pitch length $L_{0}$ is equal to 245.44 μm. The divergence angle of the quarter-pitch GIF is 0.0477 rad. In contrast, the divergence angle of SMF is 0.0987 rad.

### 3.2. Displacement Response of the Enhanced Fabry-Perot Interferometer

In this section we analyzed the displacement response of the sensor. The fiber is simplified as a slender elastic bar. The reflected light intensity decreases with the displacement increasing.

As shown in Figure 2, the tail end of the fiber was attached to a fixed fulcrum bar. As a result, the relationship between the deflection displacement *h* and the load *F* was obtained as [27]

(5) $$h=\frac{F\cdot L_{2}{}^{3}}{3E\cdot M_{z}},$$

where F is the pressure load, $L_{2}$ is the total length of the fiber interposed between the ceramic ferrule and the fulcrum bar, E is the elasticity modulus, and $M_{z}=\pi \cdot d^{4}/64=1.198\times 10^{-5}$${\mathrm{mm}}^{4}$ is the moment of inertia along z axis of the fiber [27]. Additionally, the angle β (shown in Figure 2a) between two reflectors of the FPI could be expressed as [27]

(6) $$\beta =\frac{F\cdot L_{2}{}^{2}}{2E\cdot M_{z}}\left[1-{\left(\frac{L_{2}-L_{1}}{L_{2}}\right)}^{2}\right],$$

where $L_{1}$ is the FPI cavity length. The relationship between the angle β and the displacement h was acquired from Equations (7) and (8) as:

(7) $$\beta =\frac{3}{2}\left[1-{\left(1-\frac{L_{1}}{L_{2}}\right)}^{2}\right]\frac{h}{L_{2}}\approx 3\cdot \frac{L_{1}}{L_{2}{}^{2}}\cdot h=\mu \cdot h.$$

Here, when $L_{1}<<L_{2}$, μ equal to $3\cdot L_{1}/L_{2}{}^{2}$, which is an approximation and a simplified coefficient between β and h. Moreover, $\theta_{max}$, the maximum collected angle of the ray reflected from M2 (i.e., $I_{i2}$), determines the maximum phase difference between the two reflected light (i.e., $I_{i1}$ and $I_{i2}$). It is given by [28]:

(8) $$\theta_{\max}\left(\alpha ,h\right)=\theta_{d}+\mu \cdot h\cdot \sqrt{2}cos\left(\alpha +\frac{\pi }{4}\right),$$

where α is the polar angle in the XOY plane (i.e., the fiber cross-sectional plane, as shown in the inset (b) of Figure 2), and $\theta_{d}$ is the divergence angle shown in Equation (4). The optical phase dispersion is a measure of the range of optical path lengths taken by interfering beams at various integral angles θ within the interferometer. Δφ(θ) is the maximum of the optical phase dispersion and could be expressed as [21]

(9) $$\Delta \varphi \left(\theta \right)=\Delta \varphi \left(\alpha ,h\right)=\varphi_{\max}-\varphi_{\min}=\frac{4\pi \cdot n\cdot L_{1}}{\lambda }\cdot \left[1-cos\theta_{\max}\left(\alpha ,h\right)\right]=\varphi_{0}\cdot \left[1-cos\theta_{\max}\left(\alpha ,h\right)\right].$$

Here, $\varphi_{\max}$ and $\varphi_{\min}$ are the maximum and minimum optical phase difference of the two reflected rays respectively,n is the refractive index of the air in the FPI cavity, λ is the wavelength of the light source, $\varphi_{0}$ equal to $4\pi nL_{1}/\lambda$ is the original optical phase along the fiber axis. 

The intensity of the variable reflected light is then given by [28]:

(10) $$\begin{array}{ll} \frac{I}{I_{1}}=\frac{I}{R\cdot I_{0}} & =\;\left(1+{\left(1-R\right)}^{2}\right)+\int_{\varphi_{0}-\Delta \varphi }^{\varphi_{0}}\frac{1}{\Delta \varphi \left(\alpha ,h\right)}\cdot 2\left(1-R\right)\cdot cos\varphi \\ & =\;\left(1+{\left(1-R\right)}^{2}\right)+2\left(1-R\right)\cdot \frac{1}{\pi }\cdot \left[\int_{0}^{\pi }\frac{1}{\Delta \varphi \left(\alpha ,h\right)}\int_{\varphi_{0}-\Delta \varphi }^{\varphi_{0}}cos\varphi \left(\alpha ,h\right)d\varphi d\alpha \right]\\ & =\;\left(R+{\left(1-R\right)}^{2}\right)+2\left(1-R\right)\cdot \frac{1}{\pi }\cdot \int_{0}^{\pi }\frac{sin\varphi_{0}-sin\left[\Delta \varphi \left(\alpha ,h\right)\right]}{\Delta \varphi \left(\alpha ,h\right)}d\alpha , \end{array}$$

Here, I, $I_{1}$, and $I_{0}$ are the reflected light intensity from two mirrors (M1 and M2), the reflected light from M1, and the total light intensity in the fiber, respectively. R is the reflectivity of the M1 and M2. In summary, the transmission loss could be reduced with a decreasing divergence angle, and then the fringe visibility of FPI can be improved. On the other hand, the fringe visibility of spectrum decreases with a longer FPI cavity ($L_{1}$). The greater vibration magnitude will produce larger variations of the reflected light intensity. In addition, it could be seen from Equation (9) that the bend angle (β) becomes larger in case $L_{1}$ is increased. Hence, we chose a compatible FPI cavity length of $L_{1}$ = 200 μm in the following displacement and vibration sensing experiments.

### 3.3. Natural Frequency of the Device

We analyzed the dynamic response of the device based on the previous displacement analysis. The natural frequency (NF) is calculated because of the unstable sensor performance of the device operating at this frequency, and the NF of the sensor is expressed as [19]:

(11) $$f=\frac{3.516}{2\pi }\cdot {\left(\frac{E\cdot M_{z}}{\rho \cdot A\cdot L_{3}{}^{4}}\right)}^{\frac{1}{2}},$$

where E is the modulus of elasticity, ρ is the density, $A=\pi \cdot {\left(d/2\right)}^{2}=1.227\times 10^{-3}$ ${\mathrm{mm}}^{2}$ is the cross sectional area [27], and $L_{3}$ is the total length of the sensor. The proposed sensor parameters included a Young’s modulus (E) of 72 GPa, a length ($L_{3}$) of 20 mm and a material density (ρ) 2450 kg/m^3^. As a result, the proposed FPI vibration sensor has a theoretical natural frequency of 237 Hz, and hence is suitable for detecting vibration signals.

## 4. Device Fabrication

**Figure 3:**
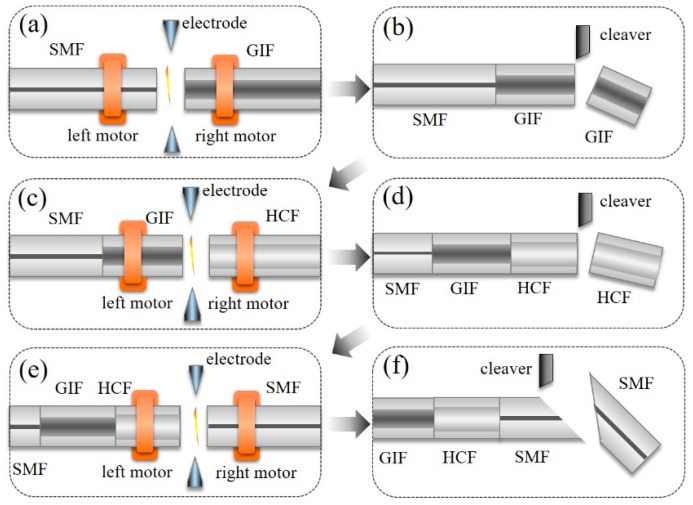
The fabrication process for the vibration sensor based on an FPI with an in-fiber collimator. (**a**) Step 1: splicing a segment of GIF to the SMF; (**b**) Step 2: cleaving the GIF with remaining length of 200 μm; (**c**) Step 3: splicing a segment of HCF to the end of GIF; (**d**) Step 4: cleaving the HCF with the remaining length of 200 μm; (**e**) Step 5: splicing a segment of SMF to the end of HCF; (**f**) Step 6: cleaving an inclined end face of the SMF with residual length of 20 mm.

The fabrication process for this FPI with an in-fiber collimator included six steps, as shown in Figure 3. In step 1, as shown in Figure 3a, a section of GIF (YOFC, 62.5/125GI0.275, Wuhan, China) was spliced with an SMF (Corning SMF-28, Corning, NY, USA) using a commercial fusion splicer (Fujikura-60S, Tokyo, Japan), and the splicer was set to a manual mode with the parameters of −35 bit in discharge intensity and 300 ms in arc time. In step 2, as shown in Figure 3b, the GIF was cleaved into a quarter-pitch length (245 μm) using a fiber cleaving system, which had an accuracy of ~±10 μm. This system consisted of two precision translation stages (Thorlabs, XR25P/M, Newton, NJ, USA), a fiber cleaver (Sumitomo FC-6S, Osaka, Japan), and an optical microscope (Sunway, Tainan, Taiwan). In step 3, as shown in Figure 3c, an HCF was spliced with the quarter-pitch GIF. In step 4, as shown in Figure 3d, the HCF was cleaved into a length of ~200 μm by the fiber cleaving system. In step 5, as shown in Figure 3e, another SMF was spliced to the flat end of the HCF. This splicing joint, i.e., the HCF-SMF interface, was used as the second reflecting surface. In step 6, as shown in Figure 3f, the fiber tail was cut to a residual length of 20 mm at an inclined angle to prevent the reflection from the end face.

**Figure 4:**
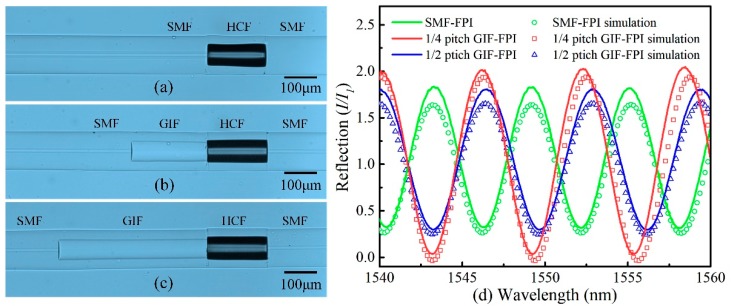
(**a**–**c**) Microscope images of an SMF FPI, a quarter-pitch (245 μm) GIF-FPI, and a half-pitch (490 μm) GIF-FPI with the same FPI cavity length of ~200 μm, respectively. (**d**) Measured and simulated reflection spectra of the SMF-FPI, a quarter-pitch GIF-FPI, and a half-pitch GIF-FPI. (Simulation parameters: Δ = 0.02, r = 31.25 μm, $\omega_{0}$ = 5 μm (in SMF), λ = 1550 nm and $n_{0}$ = 1.491, R=0.04, $L_{1}=$ 199.6, 191.8, and 198.8 μm respectively, for a SMF-FPI, a quarter-pitch GIF-FPI and a half-pitch GIF-FPI).

An in-fiber collimator based on a quarter-pitch GIF was utilized to enhance the fringe visibility and decrease the insertion loss in the FPI. A 1 × 2, 3-dB fiber coupler was used to investigate the FPI reflection spectra by connecting a broadband light source (BBS) to an optical spectrum analyzer (OSA, Yokagawa, AQ6370D, Tokyo, Japan). The side-view microscope image of the SMF-FPI, a quarter-pitch (245 μm) GIF-FPI, and a half-pitch (490 μm) GIF-FPI is shown in Figure 4a–c, respectively. The cavity lengths of the SMF-FPI, the quarter-pitch GIF-FPI, and the half-pitch GIF-FPI were almost the same (i.e., ~200 μm). Figure 4d showed the corresponding reflection spectra of these FPIs in linear coordinates. The solid green, red and blue lines represent the experimental results of an SMF-FPI, a quarter-pitch GIF-FPI, and a half-pitch GIF-FPI, respectively. It is obvious the fringe visibility in the spectrum of the quarter-pitch GIF-FPI (0.966) was much higher than that of the other two (0.702 and 0.718 for SMF-FPI and half-pitch GIF-FPI, respectively). In addition, it could be seen from Figure 4d that the simulation results (hollow circles, hollow rectangles, and hollow triangles in Figure 4d) calculated from Equation (10) agreed well with the measurement results (solid lines in Figure 4d). The results indicate the quarter-pitch GIF can be used to significantly increase the fringe visibility and reflection intensity.

## 5. Static Displacement Measurement

We investigated the static displacement response of the quarter-pitch (245 μm) GIF-FPI with a cavity length of 200 μm using the experimental setup shown in Figure 1. A narrow fulcrum bar was installed on a vertical displacement stage and used to adjust the displacement of these FPI devices on one end. The displacements could be observed and measured from the side-view images captured by an optical microscope. The reflection spectrum of the quarter-pitch (245 μm) GIF-FPI was measured using a wavelength tunable laser source and a power meter. The static displacement response was tested by moving the fulcrum bar and monitoring the spectra evolution. The resulting displacement was varied from 0 to 3000 μm. For comparison, the static displacement response of the SMF-FPI and the half-pitch (490 μm) GIF-FPI were also tested using the same method.

**Figure 5:**
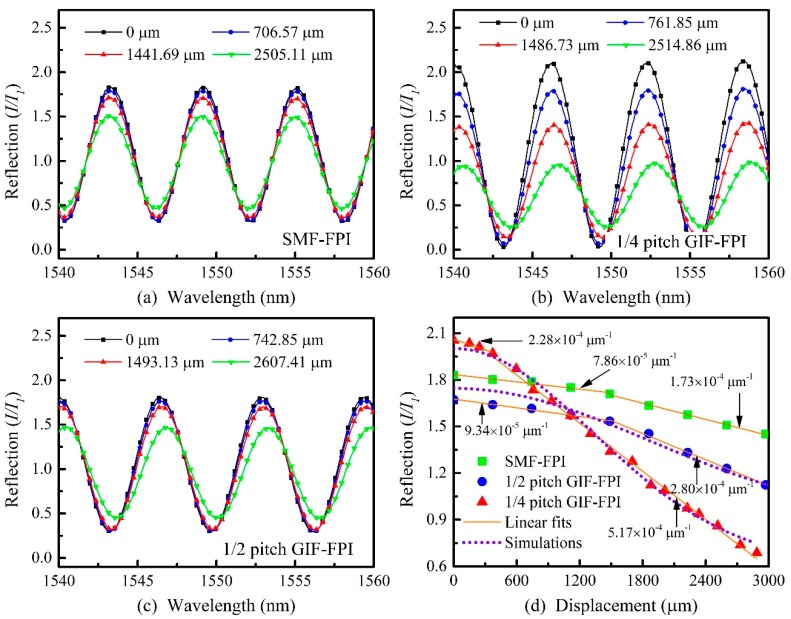
(**a**–**c**) Reflection spectra of a SMF-FPI, a quarter-pitch GIF-FPI, and a half-pitch GIF-FPI with a cavity length of 200 μm at various static displacements; (**d**) simulation results and static displacement responses of a SMF-FPI, a quarter-pitch GIF-FPI, and a half-pitch GIF-FPI with the same cavity length of 200 μm. (Simulation parameters: Δ = 0.02, r = 31.25 μm, $\omega_{0}$ = 5 μm, λ = 1550 nm and $n_{0}$ = 1.491 (GIF), $L_{2}=$ 10 mm, for a SMF-FPI, a quarter-pitch GIF-FPI and a half-pitch GIF-FPI, R=0.04 ).

Figure 5a–c demonstrate various reflection spectra for the quarter-pitch (245 μm) GIF-FPI device, the SMF-FPI, and the half-pitch (490 μm) GIF-FPI at different static displacements. It is obvious the reflection spectra for the quarter-pitch (245 μm) GIF-FPI exhibit strong dependence on the applied displacement ranging 0–3000 μm. However, the SMF-FPI and the half-pitch (490 μm) GIF-FPI showed a much weaker variation over the same displacement range. As shown in Figure 2b, the light spot reflected on M2 moves up along the *x* axis with an increasing deflection displacement on the sensor. As a result, part of the reflection cannot reenter the GIF core in the case of $\alpha <\alpha_{0}$, leading to a decrease in the reflection intensity. The quarter-pitch GIF has a much smaller divergence angle than the SMF and the half-pitch GIF, and hence is more sensitive to the variations in reflection intensity than the other two types.

The laser wavelengths were set to be 1549.10, 1552.10, and 1552.90 nm for the SMF-FPI, the quarter-pitch GIF-FPI, and the half-pitch GIF-FPI, respectively. The largest displacement response can be obtained at these wavelengths. The simulation and experimental results with different displacements of these sensors were shown in Figure 5d. The displacement sensitivity for the quarter-pitch (245 μm) GIF-FPI over different displacement ranges of 0 to 400 μm and 400 to 3000 μm were 2.28 × 10^−4^ and 5.17 × 10^−4^ μm^−1^, respectively. In contrast, the displacement sensitivity of the SMF-FPI over ranges of 0 to 1500 μm and 1500 to 3000 μm were much lower, i.e., 7.86 × 10^−5^ and 1.73 × 10^−4^ μm^−1^, respectively. Moreover, the displacement sensitivity of the half-pitch (490 μm) GIF-FPI over ranges 0–1500 μm and 1500–3000 μm were 9.34 × 10^−5^ and 2.80 × 10^−4^ μm^−1^, respectively. In addition, the experimental results agree well with the simulation results. The quarter pitch GIF-FPI has a larger fringe visibility than the SMF-FPI and the half-pitch GIF-FPI. So, the sensor has a larger displacement response than the two others.

## 6. Vibration Response

We investigated the vibration response of the quarter-pitch (245 μm) GIF-FPI, the SMF-FPI, and the half-pitch GIF-FPI with the same cavity length of ~200 μm. The experimental setup was also shown in Figure 1. The laser wavelength was set as the peak wavelength of the FPI reflection spectrum. The peak wavelength of the SMF-FPI, the quarter-pitch GIF-FPI, and the half-pitch GIF-FPI were 1549.10, 1552.10, and 1552.90 nm, respectively. The laser output power was set to 6 mW to work steadily for a long time. Amplified sinusoidal signals were applied onto the vibration generator, applying vibration signals on these FPI sensors through the fulcrum bar. The reflected optical power, which contained the vibration signal, was detected by the PD, amplified by the TIA, collected by the DAQ, and finally sent to the PC for signal processing and displaying. A PZT accelerometer was used to detect the real-time vibration for use as reference. The magnification in TIA was set to 10^3^ and the DAQ sampling rate was set to 10 kS/s.

**Figure 6:**
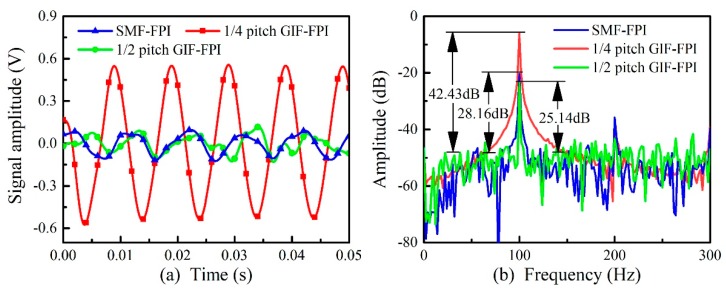
The time domain (**a**) and the frequency domain (**b**) for vibration response of an SMF-FPI, a quarter-pitch GIF-FPI and a quarter-pitch GIF-FPI with a cavity length of 200 μm at 100 Hz.

At first, we set the vibration frequency to 100 Hz and the vibration amplitude to 8.48 g. Figure 6a shows the detected vibration signals in time domain by the SMF-FPI, the quarter-pitch (245 μm) GIF-FPI, and the half-pitch (490 μm) GIF-FPI with the same cavity length of 200 μm. Moreover, fast Fourier transform (FFT) was then applied to these vibration signals and the corresponding spectra in frequency domain were shown in Figure 6b. The signal-to-noise-ratios (SNRs) for the SMF-FPI, the quarter-pitch GIF-FPI and the half-pitch GIF-FPI were 28.16, 42.43, and 25.14 dB, respectively. Hence, the minimum detectable vibration intensity was obtained, i.e., 0.33, 0.064, and 0.47 g, respectively. It is evident that the vibration signal detected by the quarter-pitch GIF-FPI sensor is significantly larger than that detected by the SMF-FPI sensor and the half-pitch GIF-FPI sensor with the same vibration acceleration (the same vibration frequency and the same vibration amplitude), while the harmonic intensities were almost the same for these three types of sensors. As a result, the employment of the quarter-pitch GIF can increase the vibration sensitivity of an FPI sensor.

**Figure 7:**
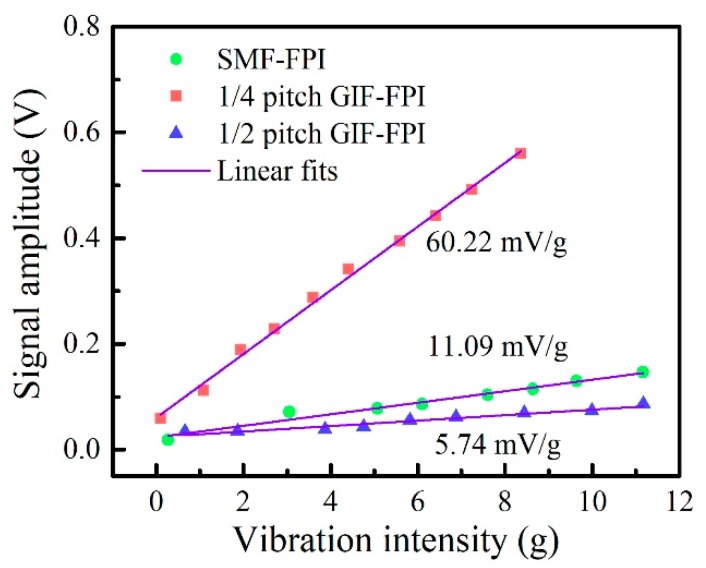
The linearity of the vibration responses of the SMF-FPI, the quarter-pitch GIF-FPI, and the half-pitch GIF-FPI with the same cavity length of 200 μm at a vibration frequency of 100 Hz.

Subsequently, we tested and recorded the linearity of these FPI vibration sensors by varying the amplitudes from 0.10 to 11.00 g at the same frequency of 100 Hz. And the results are shown in Figure 7. The acceleration sensitivities of the SMF-FPI, the quarter-pitch GIF-FPI, and the half-pitch GIF-FPI were 11.09, 60.22, and 5.74 mV/g, respectively. It can be seen that the acceleration sensitivity of the quarter-pitch GIF-FPI was more than five times higher than that of the SMF-FPI and the half-pitch GIF-FPI. The R-square value of the linear fit for the quarter-pitch GIF-FPI is 0.996, showing a high linearity of the vibration response. The other R-square values of the linear fits for the SMF-FPI and the half-pitch GIF-FPI are 0.96 and 0.94, respectively.

**Figure 8:**
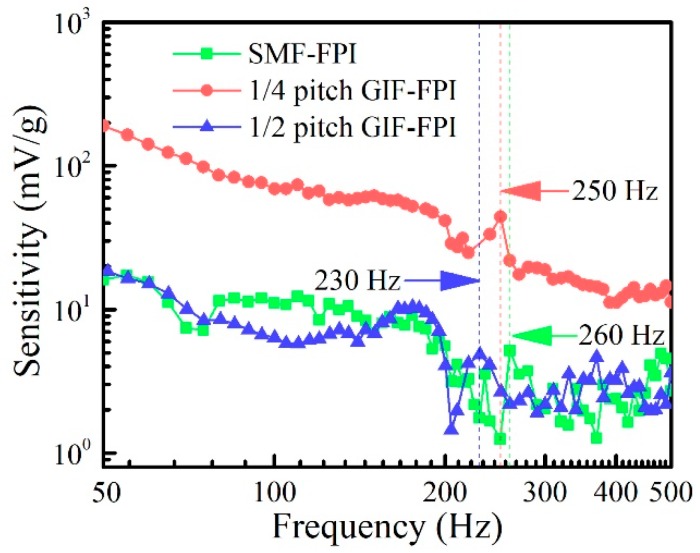
The frequency responses of the vibration sensors based on the SMF-FPI, the quarter-pitch GIF-FPI, and the half-pitch GIF-FPI with same cavity length of 200 μm. Different vibration frequency ranging from 40 Hz to 500 Hz was investigated.

Finally, we tested the frequency response of these FPI vibration sensors using various vibration frequencies ranging from 40 to 500 Hz. The results are shown in Figure 8, where the blue, red, and green dash-dot lines represent the SMF-FPI, the quarter-pitch GIF-FPI, and the half-pitch GIF-FPI, respectively. It is obvious that the quarter-pitch GIF-FPI sensor has higher acceleration sensitivities than those of the other two types of FPI sensors at the same vibration frequency ranging from 40 to 500 Hz. The frequency response curves demonstrate that the SMF-FPI, the quarter-pitch GIF-FPI, and the half-pitch GIF-FPI have a natural frequency of 260, 250, and 230 Hz, respectively. A relatively flat frequency response was obtained in the frequency range from 80 to 200 Hz. The acceleration sensitivity decreased gradually as the vibration frequency exceeded the natural frequency. It should be noted that the vibration measurement results are in accordance with the theoretical expectations and the static displacement experimental results. Moreover, the higher vibration sensitivity of the quarter-pitch GIF-FPI than that of the half-pitch GIF-FPI and SMF-FPI may result from its smaller divergence angle. These results demonstrate that the quarter-pitch GIF-FPI is capable of detecting the vibrational signal at frequency range of 80 to 200 Hz.

## 7. Conclusions

A compact FPI fiber sensor with an in-fiber collimator has been investigated for both static displacement and dynamic vibration sensing. The device consists of a quarter-pitch GIF spliced with a section of HCF, which was integrated between two SMFs. The static and dynamic response of the device were investigated. Results showed that the quarter-pitch GIF-FPI achieved the highest static displacement sensitivity of 5.17 × 10^−4^ μm^−1^, which was higher than that of the SMF-FPI (1.73 × 10^−4^ μm^−1^). The vibration acceleration sensitivity of the quarter-pitch GIF-FPI was 60.22 mV/g at 100 Hz, which was significantly higher than the sensitivity of the SMF-FPI (i.e., 11.09 mV/g at 100 Hz). The quarter-pitch GIF-FPI exhibited a responsibility one order of magnitude higher than the SMF-FPI at frequencies ranging from 40 to 200 Hz. As such, the proposed fiber vibration sensor could be used for hot fluid dynamics, construction safety inspection or the development of new energy sources.

## References

[1] Colchester R.J., Zhang E.Z., Mosse C.A., Beard P.C., Papakonstantinou I., Desjardins A.E. (2015). Broadband miniature optical ultrasound probe for high resolution vascular tissue imaging. Biomed. Opt. Express.

[2] Manuel R.M., Shlyagin M.G., Miridonov S.V., Meyer J. (2012). Vibration disturbance localization Using a serial array of identical low-finesse fiber Fabry-Perot interferometers. IEEE Sens. J..

[3] Preisser S., Rohringer W., Liu M., Kollmann C., Zotter S., Fischer B., Drexler W. (2016). All-optical highly sensitive akinetic sensor for ultrasound detection and photoacoustic imaging. Biomed. Opt. Express.

[4] Pullteap S., Seat H.C. (2014). An extrinsic fiber Fabry-Perot interferometer for dynamic displacement measurement. Photonic Sens..

[5] Wada A., Tanaka S., Takahashi N. (2012). Optical fiber vibration sensor using FBG Fabry–Perot interferometer with wavelength scanning and Fourier analysis. IEEE Sens. J..

[6] Xu X., He J., Hou M., Liu S., Bai Z., Wang Y., Liao C., Ouyang Z., Wang Y. (2018). A miniature fiber collimator for highly sensitive bend measurements. J. Lightwave Technol..

[7] Zhang Z., He J., Dong Q., Bai Z., Liao C., Wang Y., Liu S., Guo K., Wang Y. (2018). Diaphragm-free gas-pressure sensor probe based on hollow-core photonic bandgap fiber. Opt. Lett..

[8] Liao C., Liu S., Xu L., Wang C., Wang Y., Li Z., Wang Q., Wang D.N. (2014). Sub-micron silica diaphragm-based fiber-tip Fabry-Perot interferometer for pressure measurement. Opt. Lett..

[9] Xu X., Wang Y., Liu S., Liao C., He J., Lian J., Wang Y. (2017). Growth dynamics of ZnO nanowire on a fiber-tip air bubble. Opt. Mater. Express.

[10] Majchrowicz D., Hirsch M., Wierzba P., Bechelany M., Viter R., Jedrzejewska-Szczerska M. (2016). Application of thin ZnO ALD layers in fiber-optic Fabry-Perot sensing interferometers. Sensors.

[11] Barnes J., Li S., Goyal A., Abolmaesumi P., Mousavi P., Loock H. (2018). Broadband vibration detection in tissue phantoms using a fiber Fabry–Perot cavity. IEEE Trans. Biomed. Eng..

[12] Hirsch M., Majchrowicz D., Wierzba P., Weber M., Bechelany M., Jedrzejewska-Szczerska M. (2017). Low-coherence interferometric fiber-optic sensors with potential applications as biosensors. Sensors.

[13] Wu S., Wang L., Chen X., Zhou B. (2018). Flexible optical fiber Fabry-Perot interferometer based acoustic and mechanical vibration sensor. J. Lightwave Technol..

[14] Zhang Z., Liao C., Tang J., Bai Z., Guo K., Hou M., He J., Wang Y., Liu S., Zhang F. (2017). High-sensitivity gas-pressure sensor based on fiber-tip PVC diaphragm Fabry–Pérot interferometer. J. Lightwave Technol..

[15] Nishino Z.T., Chen K., Gupta N. (2014). Power modulation-based optical sensor for high-sensitivity vibration measurements. IEEE Sens. J..

[16] Bai Z., Gao S., Deng M., Zhang Z., Li M., Zhang F., Liao C., Wang Y., Wang Y. (2017). Bidirectional bend sensor employing a microfiber-assisted U-shaped Fabry-Perot cavity. IEEE Photonics J..

[17] Takamori A., Araya A., Morii W., Telada S., Uchiyama T., Ohashi M. (2014). A 100-m Fabry–Pérot cavity with automatic alignment controls for long-term observations of earth’s strain. Technologies.

[18] Gardner D., Hofler T., Baker S., Yarber R., Garrett S. (1987). A fiber-optic interferometric seismometer. J. Lightwave Technol..

[19] Lopez-Higuera J.M., Morante M.A., Cobo A. (1997). Simple low-frequency optical fiber accelerometer with large rotating machine monitoring applications. J. Lightwave Technol..

[20] Kamenev O.T., Kulchin Y.N., Petrov Y.S., Khiznyak R.V., Romashko R.V. (2016). Fiber-optic seismometer on the basis of Mach-Zehnder interferometer. Sensor. Actuators A Phys..

[21] Li T., Shi C., Tan Y., Li R., Zhou Z., Ren H. (2017). A diaphragm type fiber Bragg grating vibration sensor based on transverse property of optical fiber with temperature compensation. IEEE Sens. J..

[22] Li W., Zhang J. (2018). Distributed weak fiber Bragg grating vibration sensing system based on 3 × 3 fiber coupler. Photonic Sens..

[23] Wang J., Wei L., Li R., Liu Q., Yu L. (2018). A fiber Bragg grating based torsional vibration sensor for rotating machinery. Sensors.

[24] Zhang Y., Li Y., Wei T., Lan X., Huang Y., Chen G., Xiao H. (2010). Fringe visibility enhanced extrinsic Fabry–Perot interferometer using a graded index fiber collimator. IEEE Photonics J..

[25] Wang S.H., Tay C.J., Quan C., Shang H.M. (2001). Study of collimating laser diode beam by a graded-index optical fibre. Optik.

[26] Ma C., Dong B., Gong J., Wang A. (2011). Decoding the spectra of low-finesse extrinsic optical fiber Fabry-Perot interferometers. Opt Express.

[27] Ghavami P. (2015). Mechanics of Materials: An Introduction to Engineering Technology.

[28] Pérennès F., Beard P.C., Mills T.N. (1999). Analysis of a low-finesse Fabry–Perot sensing interferometer illuminated by a multimode optical fiber. Appl. Opt..

